# Large dataset on Fourier transform near infrared (FT-NIR) spectroscopy of green and roasted specialty coffee: Preprocessed infrared spectra and sensory scores for machine learning-based quality monitoring

**DOI:** 10.1016/j.dib.2025.111609

**Published:** 2025-05-01

**Authors:** Andrés F. Bahamón-Monje, Ever M. Morales-Angulo, Gentil A. Collazos-Escobar, Nelson Gutiérrez-Guzmán

**Affiliations:** a Centro Surcolombiano de Investigación en Café (CESURCAFÉ), Departamento de Ingeniería Agrícola, Universidad Surcolombiana, Neiva-Huila 410001, Colombia; b Departamento de Ingeniería Agroindustrial, Facultad de Ingeniería, Universidad Surcolombiana, Neiva, Huila, Colombia; c Grupo de Análisis y Simulación de Procesos Agroalimentarios (ASPA), Instituto Universitario de Ingeniería de Alimentos–FoodUPV, Universitat Politècnica de València, Camí de Vera s/n, Edificio 3F, València 46022, Spain

**Keywords:** Spectral analysis, Supervised machine learning, Quality assessment, Chemometric models, Sensory attributes

## Abstract

A comprehensive dataset on FT-NIR spectra for green and roasted specialty coffee is critically needed to advance predictive models for sensory quality assessment. To address this need, the NIR spectra of green and roasted specialty coffee were obtained using cutting-edge FT-NIR spectroscopy, while sensory quality analysis for roasted coffee beans followed the standardized protocol validated by the Specialty Coffee Association (SCA). This approach was designed to establish a robust dataset for calibrating machine learning-based predictive models of coffee cup quality. FT-NIR spectra were acquired using a Spectrum Two N-FT-NIR Spectrometer equipped with a high-resolution Indium Gallium Arsenide (InGaAs) detector, operating in diffuse reflectance mode. Spectral data were collected over a wavelength range of 12,000 to 4,000 cm^–1^ with a spectral resolution of 8 cm^–1^, an interval of 4 cm^–1^, and 64 accumulated scans per sample. The dataset includes both raw and preprocessed FT-NIR spectra, incorporating baseline correction, Standard Normal Variate (SNV), Multiplicative Scatter Correction (MSC), as well as first and second derivatives to enhance spectral interpretation. One of the main strengths of this dataset is its ability to facilitate non-destructive quality assessment of roasted coffee using both green and roasted FT-NIR spectra. Furthermore, it also enables the prediction of roasted coffee sensory quality based on green coffee spectra, potentially reducing the need for roasting during quality evaluation and streamlining batch screening processes. This dataset enhances research on coffee quality, chemical marker identification, and roasting optimization, supporting both scientific and industrial applications. The dataset is structured into Excel files, systematically organized by processed samples and their replicates, providing a valuable framework for further analysis, model development, and calibration of multivariate statistical models.

Specifications Table

**Table:** 

Subject	Food science and technology
Specific subject area	Food technology, Food engineering, Food Science
Type of data	Excel files (Raw, baseline, SNV, MSC, first derivative and second derivative FT-NIR spectra, and sensory quality scores). Figures (Raw and preprocessed FT-NIR spectra of green and roasted coffee as a function of sensory quality score, and experimental procedure for dataset acquisition).
Data collection	FT-NIR (Fourier transform Near-Infrared spectroscopy) and sensory analysis based on Coffee Association methodology (SCA) protocol.
Data source location	The experimental dataset described in this study has been obtained in the Centro Surcolombiano de Investigación en Café (CESURCAFÉ) from the Universidad Surcolombiana, Neiva-Huila, Colombia.
Data accessibility	Repository name: Mendeley data Data identification number: 10.17632/nz2fr76trm.3 Direct URL to data: https://data.mendeley.com/datasets/nz2fr76trm/3
Related research article	None.

## 1 Value of the Data

• This dataset offers both raw and preprocessed FT-NIR spectra of green and roasted specialty coffee, making it an essential information for researchers exploring advanced analytical techniques in coffee science. It provides valuable data for studies on coffee quality, authenticity, and sensory evaluation, serving as a powerful tool for the scientific community.
• The dataset is reliable for the development, validation, and comparison of machine learning models designed to predict coffee sensory quality. It is particularly beneficial for researchers applying data-driven methodologies in agricultural and food sciences, facilitating progress in automated quality assessment and decision-making.
• By providing both raw and preprocessed FT-NIR spectra, this dataset enhances the understanding of spectroscopic patterns in green and roasted coffee. It reveals insights into spectral variability and its correlations with key quality indicators, contributing to a deeper comprehension of coffee’s chemical and sensory characteristics.
• The dataset promotes reproducibility and standardization in coffee quality research by offering preprocessed, consistently formatted spectra that ensure compatibility with a wide array of analytical and computational tools, streamlining workflows for researchers and industry professionals alike.
• This dataset enables the identification of functional groups in the infrared spectra of green coffee beans, which can be correlated with sensory attributes in roasted coffee. By leveraging machine learning-based models, an intelligent system can be developed to screen incoming batches of green coffee based on predicted quality, thereby selecting only high-quality samples that meet the required standards for roasting. Thus, it facilitates a rapid and non-destructive quality assessment, enhancing decision-making efficiency in both industrial and research settings.
• The dataset also provides key insights into spectral changes throughout the roasting process. Researchers and industry experts can leverage these insights to develop advanced tools for real-time monitoring and optimization of roasting parameters, ultimately supporting consistent achievement of desired sensory qualities and enhancing overall roasting efficiency.

## 2 Background

Coffee is one of the most widely consumed beverages globally and a critical agricultural commodity, deeply intertwined with cultural, social, and economic practices [1]. The global coffee industry is valued at billions of dollars annually, supporting the livelihoods of millions, particularly in tropical regions where coffee production is a cornerstone of economic stability [2]. The demand for high-quality coffee, particularly specialty coffee, has surged in recent years, driven by consumer preferences for premium flavor profiles, traceability, and sustainable sourcing [3]. In the global coffee industry, sensory evaluation plays a pivotal role in determining the quality and market value of specialty coffee. However, the traditional sensory cupping protocol, widely regarded as the gold standard, is highly labor-intensive, requiring significant time, specialized equipment, and trained personnel to evaluate each sample [4]. For coffee producers and traders, this translates into considerable economic and operational costs, particularly when assessing large volumes of coffee [5]. To address these challenges, there is an urgent need for real-time, non-invasive, non-destructive, and computationally efficient tools capable of predicting coffee quality both before and after roasting. Such tools would not only streamline the decision-making process but also reduce costs and improve scalability in the sensory analysis of coffee. In this sense, this dataset was compiled to support the development of predictive models that integrate machine learning with Fourier Transform pre-processed Near Infrared (FT-NIR) spectral data. By focusing on green and roasted specialty coffee, it offers a valuable basis for producers and researchers seeking to implement mathematical tools for quality assessment. This approach has the potential to revolutionize quality control processes in the coffee industry, enabling producers and traders to make informed decisions efficiently and cost-effectively.

## 3 Data Description

**Fig. 1:**
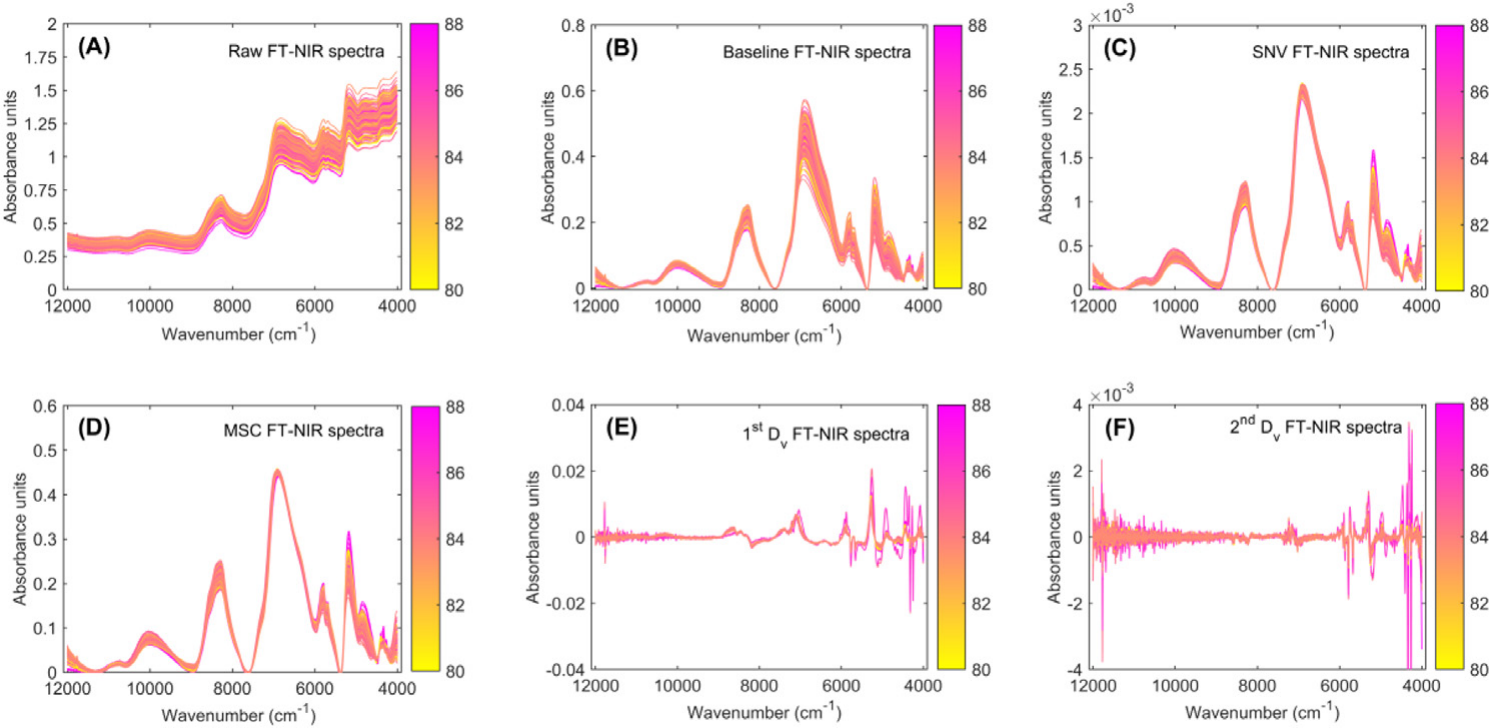
Preprocessing of Fourier Transform Near-Infrared (FT-NIR) spectra for specialty green coffee. The raw FT-NIR spectra (A), baseline correction (B), Standard Normal Variate (SNV; C), Multiplicative Scatter Correction (MSC; D), first derivative (1^st^ D_v_; E) and second derivative (2^nd^ D_v_; F) of FT-NIR preprocessed spectra. The FT-NIR spectra are color-coded according to their sensory quality scores, with the corresponding color gradient indicated by a color bar.

**Fig. 2:**
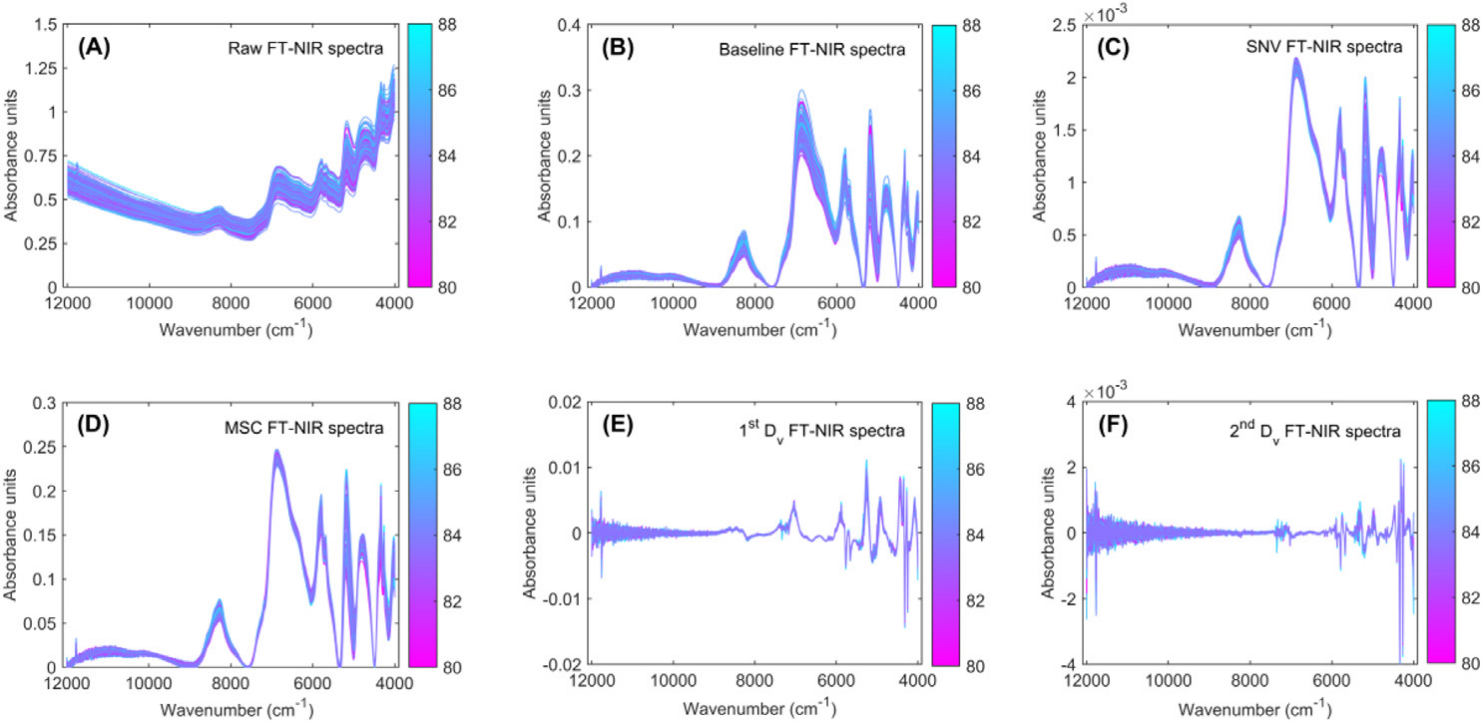
Preprocessing of Fourier Transform Near-Infrared (FT-NIR) spectra for specialty roasted coffee. The raw FT-NIR spectra (A), baseline correction (B), Standard Normal Variate (SNV; C), Multiplicative Scatter Correction (MSC; D), first derivative (1^st^ D_v_; E) and second derivative (2^nd^ D_v_; F) of FT-NIR preprocessed spectra. The FT-NIR spectra are color-coded according to their sensory quality scores, with the corresponding color gradient indicated by a color bar.

**Fig. 3:**
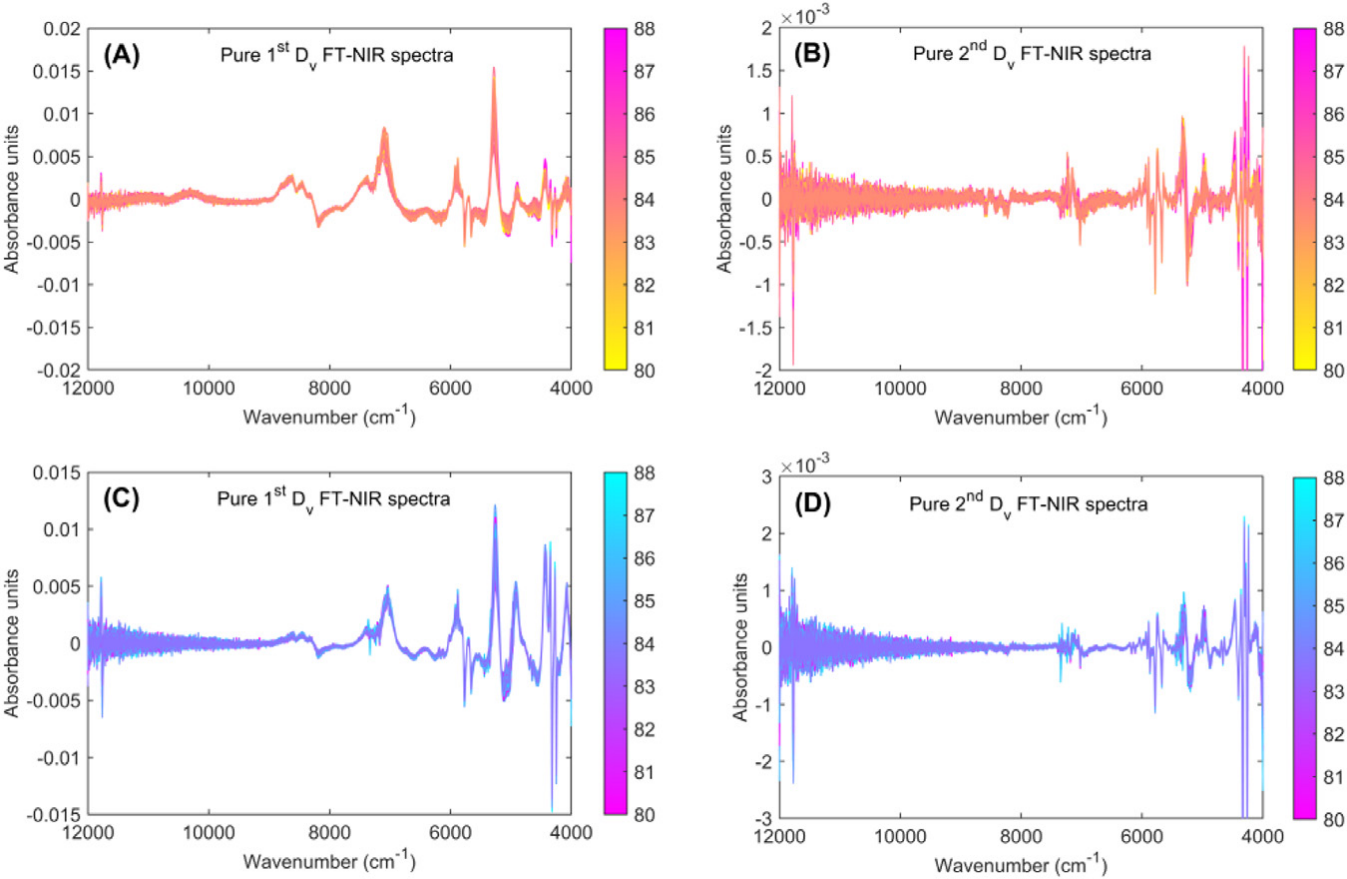
Preprocessing of Fourier Transform Near-Infrared (FT-NIR) spectra for specialty green and roasted coffee. The raw FT-NIR spectra was preprocessed by applying the first derivative (1^st^ D_v_; A) and second derivative (2^nd^ D_v_; B) for green coffee beans, and first derivative (1^st^ D_v_; C) and second derivative (2^nd^ D_v_; D) for roasted coffee beans.

The experimental dataset on FT-NIR spectra and sensory quality scores was compiled in seventeen Excel files, each of which is described in detail below. The raw, baseline, SNV, MSC, first derivative, second derivative and pure first and second derivative FT-NIR spectra of both green and roasted coffee, and the sensory quality scores for roasted coffee are illustrated in Fig. 1, Fig. 2, Fig. 3. The raw FT-NIR spectra of green and roasted coffee were preprocessed by baseline, SNV, MSC first derivative and second derivative [6] using the R statistical software (version 4.2.3, 2023, R statistics, St. Louis, MO, USA) via the *ChemoSpec* R-function [7].

All Excel data files contain spectral data obtained from FT-NIR spectroscopy, covering the wavenumber values ranging from 1200 to 4000 cm^–1^, which corresponds to the near-infrared region of the electromagnetic spectrum typically used in FT-NIR spectroscopy. Each dataset includes measurements for 64 green coffee samples and 64 roasted coffee samples, analyzed in triplicate (64 samples × 3 replicates), resulting in a total of 192 spectra per dataset. The datasets are structured as matrices, where rows represent specific wavenumbers (measured in cm^–1^), and columns correspond to absorbance values for each sample and its replicates. These datasets enable the assessment of precision, variability, and chemical composition of green and roasted coffee samples at different spectral preprocessing stages. For further details, see the EXPERIMENTAL DESIGN, MATERIALS AND METHODS section, which describes the experimental design and methods used for sample preparation and data acquisition.

**RawSpectra_GreenCoffee** and **RawSpectra_RoastedCoffee:** Contain raw FT-NIR spectral data for green and roasted coffee samples (Fig. 1A and Fig. 2A, respectively).

**BaselineCorrection_GreenCoffee** and **BaselineCorrection_RoastedCoffee:** Include baseline-corrected FT-NIR spectra for green and roasted coffee samples (Fig. 1B and Fig. 2B, respectively).

**AreaNormalization_GreenCoffee** and **AreaNormalization_RoastedCoffee**: Contain FT-NIR spectra normalized by area using the SNV method for green and roasted coffee samples (Fig. 1C and Fig. 2C, respectively).

**MultiplicativeScatterCorrection_GreenCoffee** and **MultiplicativeScatterCorrection_RoastedCoffee:** Contain FT-NIR spectra of green and roasted coffee samples preprocessed with MSC (Fig. 1D and Fig. 2D, respectively).

**FirstDerivative_GreenCoffee** and **FirstDerivative_RoastedCoffee:** Contain FT-NIR spectra of green and roasted coffee samples preprocessed with the first derivative (Fig. 1E and Fig. 2E, respectively).

**SecondDerivative_GreenCoffee** and **SecondDerivative_RoastedCoffee:** Include FT-NIR spectra of green and roasted coffee samples preprocessed with the second derivative (Fig. 1F and Fig. 2F, respectively).

**PureFirstDerivative_GreenCoffee** and **PureSecondDerivative_GreenCoffee** datasets contain spectral data for 64 green coffee samples, each analyzed in triplicate, resulting in 192 spectra (64 samples × 3 replicates per Excel file). The raw spectra (Fig. 1A) were directly preprocessed using the first derivative (Fig. 3A) and second derivative (Fig. 3B). This preprocessing was performed to obtain the pure first and second derivative spectra of the green coffee samples.

**PureFirstDerivative_RoastedCoffee** and **PureSecondDerivative_RoastedCoffee** datasets contain spectral data for 64 roasted coffee samples, each analyzed in triplicate, resulting in 192 spectra (64 samples × 3 replicates per Excel file). The raw spectra (Fig. 2A) were directly preprocessed using the first derivative (Fig. 3C) and second derivative (Fig. 3D). This preprocessing was performed to obtain the pure first and second derivative spectra of the roasted coffee samples.

**SensoryQuality_RoastedCoffee:** This dataset includes sensory evaluation scores for 64 roasted coffee samples, each analyzed in triplicate. The dataset is structured with the first column representing the sample identification, the second column corresponding to the replicate number, and the third column containing the overall sensory quality score for each sample. The sensory quality score serves as the response variable, reflecting the overall perceived quality of the coffee beans based on expert sensory evaluations (see the EXPERIMENTAL DESIGN, MATERIALS AND METHODS section). These scores are critical for developing and calibrating predictive models of sensory quality. The primary use of the dataset is for machine learning model calibration, where the sensory quality scores can be modeled using raw, baseline, SNV, MSC, first derivative, and second derivative FT-NIR spectra. This approach aims to calibrate a robust multivariate machine learning-based model for predicting sensory attributes of roasted coffee. The dataset enables the development of such predictive models that could be used for non-destructive quality monitoring and control, making it a valuable tool for quality assessment in both industrial and research settings. Designed to bridge the gap between sensory evaluation and spectroscopic analysis, this dataset provides essential data for the development of automated systems capable of predicting sensory quality from NIR spectra, offering efficiency and consistency in quality control processes [8].

An important advantage of this dataset is that it provides the ability to predict the sensory quality of roasted coffee using the FT-NIR spectra of green coffee beans. This capability enables precise quality estimations without the need to roast a sample, significantly saving time and resources. By leveraging the spectral data from green coffee, researchers and industry professionals can perform accurate sensory quality assessments at the green coffee stage, prior to roasting. This non-destructive predictive approach is particularly valuable for coffee producers, as it allows for early screening of incoming batches, streamlining the quality control process. By implementing this strategy in a batch screening system, producers can make quick, data-driven decisions about the quality of green coffee before roasting, minimizing the need for time-consuming and resource-intensive sensory evaluation methods. Furthermore, this method can help optimize the roasting process itself. By understanding the quality of green coffee in advance, producers can tailor roasting parameters better to achieve desired sensory characteristics, improving consistency and efficiency in production. This approach thus not only accelerates quality assurance but also contributes to overall operational cost reduction in the coffee industry.

Future research should integrate samples from diverse geographical regions and climatic conditions while also considering key variables such as coffee agricultural practices, fermentation methods, drying techniques, and roasting parameters within the dataset. By incorporating these factors, predictive models can be significantly strengthened, capturing the intricate variability inherent in coffee production systems and improving their accuracy and applicability.

## 4 Experimental Design, Materials and Methods

**Fig. 4:**
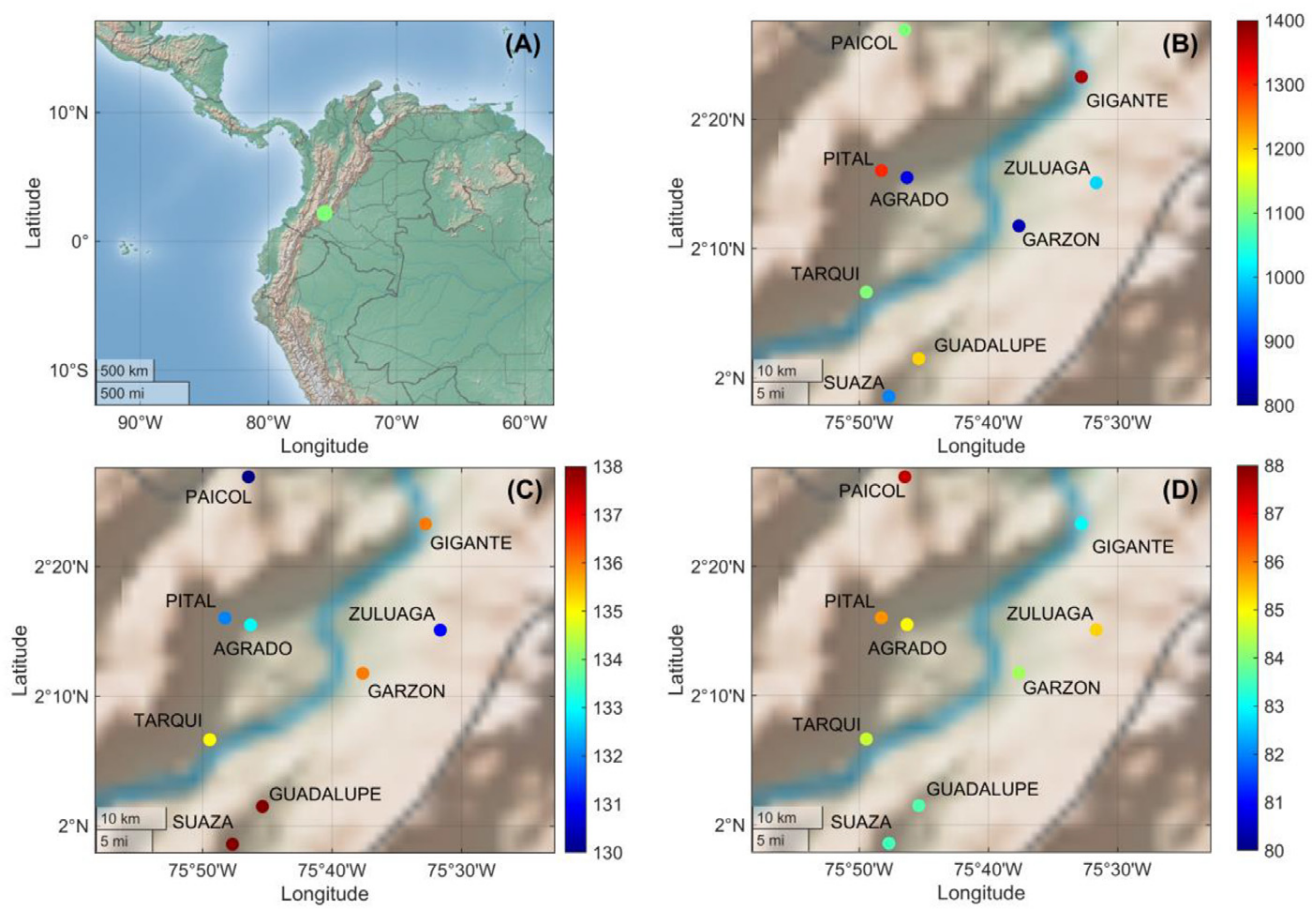
Geographic distribution and environmental characteristics of coffee sample origins from the Huila region, Colombia. The location of the Huila region within Colombia (A), geographical coordinates of sampled coffee-growing areas, with points colored according to elevation (meters above sea level) (B), geographical coordinates of sampled coffee-growing areas, with points colored based on the average annual rainfall of the sampling locations (C) and geographical distribution of coffee samples, with points colored according to their sensory quality scores (D).

Fresh coffee samples (*Coffea arabica* L., n=64) were harvested from different growing areas in the municipalities of Garzón, Gigante, Agrado, Zuluaga, Tarqui, Pital, Guadalupe, and Suaza in the Huila region of Colombia (Fig. 4). The inclusion of environmental variables such as altitude, rainfall, and geographic location is crucial in the development of robust ML models for predicting coffee quality, as these factors could significantly influence the chemical composition, growth conditions, and sensory attributes of coffee.

**Fig. 5:**
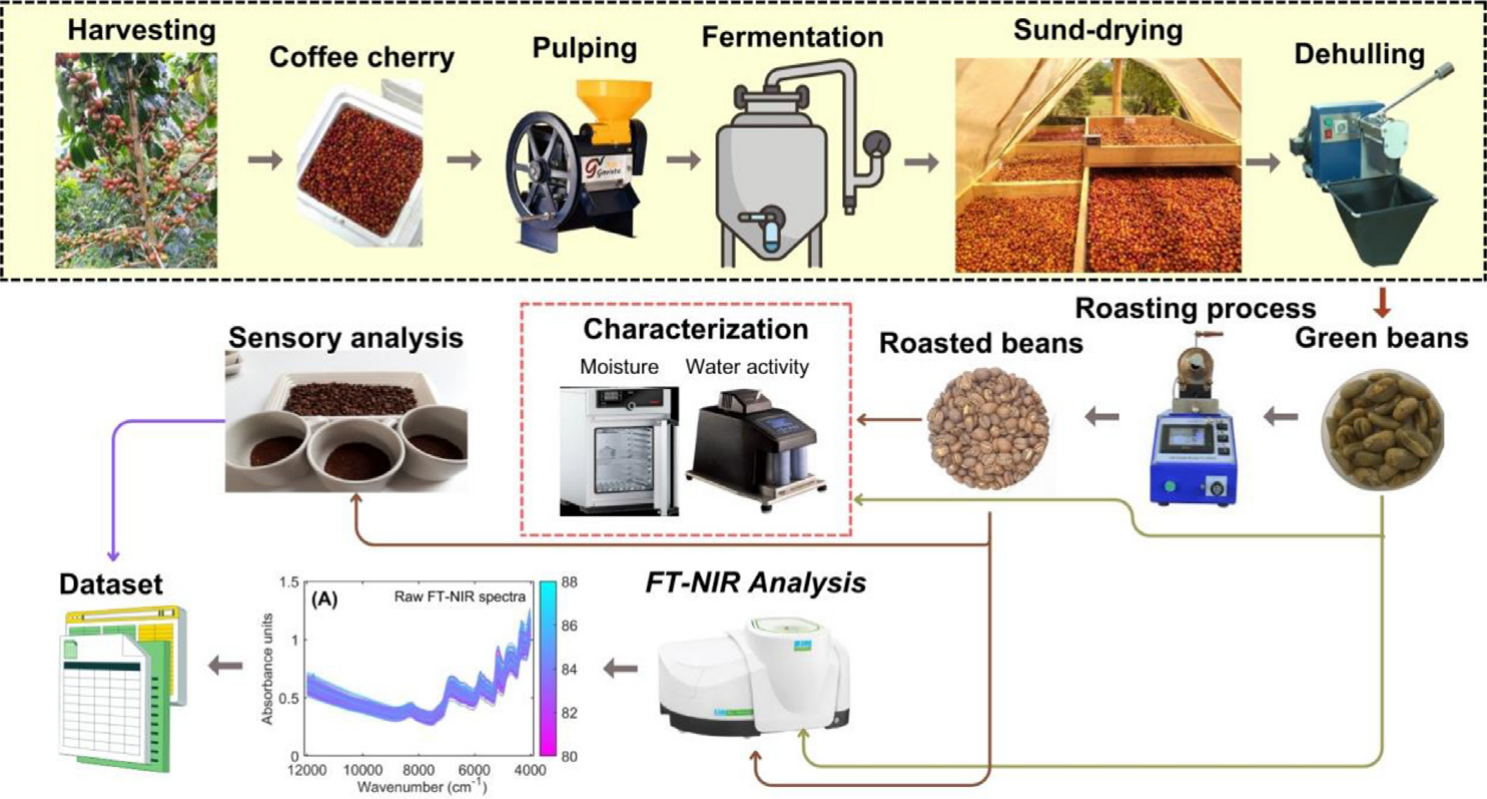
Flowchart illustrating the experimental procedure for acquiring the dataset of Fourier Transform Near-Infrared (FT-NIR) spectra and sensory quality scores in green and roasted specialty coffee samples.

The coffee samples were processed by the wet method [9] and sun-dried until they reached a moisture content of 10-12% wet basis. The final point of sun-drying was defined using a handheld portable grain moisture tester (Kett PM−450, Science of Sensing, Japan). Dried coffee samples were analyzed in the Centro Surcolombiano de Investigación en Café (CESURCAFÉ, Neiva-Huila, Colombia). Dried coffee samples were dehulled in a hulling machine (ING-C-250, Ingesec, Colombia) to remove the parchment peel of dried coffee and obtain the so-called green coffee beans (Fig. 5). Then, the green beans were roasted using laboratory rotary equipment (TC-150R, Quantik, Colombia). The roasting process was conducted following different steps. Firstly, a preheating stage was applied, where the rotary system was heated to 150°C for 10 minutes before loading the coffee beans. Once preheating was complete, the system was heated to 185 ± 2°C, followed by a slight cooling adjustment to 178 ± 2°C before introducing the coffee samples into the roasting chamber. During roasting, temperature monitoring was performed at 5-second intervals to ensure process control. After 3.5 minutes, the heating power level was increased from 10% to 60% to accelerate the roasting reaction. The roasting process was completed when the total roasting time reached 10 ± 0.5 minutes, with a final recorded temperature of 183 ± 2°C. In every roasting batch, two cracks in the coffee beans serve to indicate bean expansion and the quality of the process.

The green and roasted coffee samples were initially characterized by determining the moisture content and water activity. To achieve this, the moisture content of the coffee beans was determined using the gravimetric method. A 5 g sample was placed in a laboratory oven (UF55, Memmert GmbH + Co. KG, Schwabach, Germany) and heated at 105 ± 1°C for approximately 24 hours [10]. The water activity of the coffee samples was measured using a vapor sorption analyzer (VSA Aqualab, Decagon Devices, Inc. Pullman, WA).

FT-NIR spectra were acquired for both green and roasted coffee bean samples using a Spectrum Two N-FT-NIR spectrometer (PerkinElmer, Inc., USA). To enhance the precision of spectral acquisition, a petri dish containing the coffee samples was placed on a rotational accessory to ensure an even scan. The spectrometer operated in diffuse reflectance mode and was equipped with a high-resolution InGaAs detector. FT-NIR spectra were recorded within a wavelength range of 12,000 to 4000 cm⁻¹ with a data interval of 4 cm⁻¹ and a spectral resolution of 8 cm⁻¹. To improve the reliability of the data, each measurement was performed in triplicate (see DATA DESCRIPTION section), generating three spectral replicates per sample. Each acquired spectrum contained 2000 data points (wavenumber), ensuring a high level of precision and consistency across all measurements.

After all laboratory analyses, the labeled and vacuum-sealed roasted coffee samples were sent to the Coffee Processing Plant of the Central Cooperative of Coffee Growers (COOCENTRAL), located at Kilometer 2 on the road to the municipality of Agrado, Huila, Colombia, for sensory analysis. The SCA sensory analysis methodology was employed to evaluate the roasted coffee bean samples. This standardized system assesses coffee quality based on key sensory attributes, including fragrance/aroma, flavor, aftertaste, acidity, body, balance, uniformity, cleanliness, sweetness, and overall impression. The evaluation was conducted by five expert cuppers, following the official SCA cupping protocol. Each attribute was scored on a 100-point scale, where a score of 80 or above qualifies as specialty coffee. Strict guidelines regarding sample preparation, water quality, and cupping procedures were adhered to, ensuring objective and reproducible results [11]. This methodology allowed for a comprehensive assessment of the sensory profile of the analyzed samples.

## Limitations

None.

## Ethics Statement

The dataset acquired in this study did not involve human subjects, animal experiments, or data obtained from social media platforms.

## CRediT authorship contribution statement

**Andrés F. Bahamón-Monje:** Conceptualization, Methodology, Software, Data curation, Writing – original draft. **Ever M. Morales-Angulo:** Methodology, Software, Data curation, Visualization, Writing – original draft. **Gentil A. Collazos-Escobar:** Conceptualization, Methodology, Software, Data curation, Visualization, Writing – original draft. **Nelson Gutiérrez-Guzmán:** Conceptualization, Supervision, Writing – review & editing.

## Data Availability

Mendeley DataFourier Transform Near Infrared (FT-NIR) spectra and sensory scores in green and roasted specialty coffee for machine learning-based quality monitoring (Original data).
